# Association of Circulating GDF-15 with Fetal Growth in Gestational Diabetes

**DOI:** 10.3390/jcm14082764

**Published:** 2025-04-17

**Authors:** Tiziana Filardi, Enrico Bleve, Valentina Viggiani, Paola Galoppi, Giuseppe Rizzo, Stefania Gorini, Massimiliano Caprio, Antonio Angeloni, Susanna Morano, Emanuela Anastasi

**Affiliations:** 1 Department for the Promotion of Human Sciences and Quality of Life, San Raffaele Roma Open University, Via di Val Cannuta, 247, 00166 Rome, Italy; tiziana.filardi@uniroma5.it (T.F.); stefania.gorini@uniroma5.it (S.G.); massimiliano.caprio@uniroma5.it (M.C.); 2 Department of Experimental Medicine, “Sapienza” University, Viale Regina Elena 324, 00161 Rome, Italy; enrico.bleve@uniroma1.it (E.B.); antonio.angeloni@uniroma1.it (A.A.); emanuela.anastasi@uniroma1.it (E.A.); 3 Department of Molecular Medicine, “Sapienza” University, Viale Regina Elena 291, 00161 Rome, Italy; valentina.viggiani@uniroma1.it; 4 Maternal and Child Health and Urological Sciences, “Sapienza” University, Viale Regina Elena 324, 00161 Rome, Italy; paola.galoppi@uniroma1.it (P.G.); giuseppe.rizzo@uniroma1.it (G.R.); 5 Laboratory of Cardiovascular Endocrinology, San Raffaele Research Institute, IRCCS San Raffaele Roma, Via di Val Cannuta, 247, 00166 Rome, Italy

**Keywords:** growth differentiation factor-15, GDF-15, gestational diabetes mellitus, perinatal outcomes, small dense low density lipoprotein cholesterol, interleukin-6, soluble urokinase plasminogen activator receptor, fetal growth, inflammation, pregnancy

## Abstract

**Background/Objectives**: Growth differentiation factor-15 (GDF-15) is a component of the transforming growth factor beta (TGF-β) family that may act as regulator of inflammation. A possible protective role of GDF-15 against glucose alterations has been hypothesized. The aim of this pilot study was to evaluate the relationship between a circulating concentration of GDF-15 and metabolic/inflammatory parameters, as well as with adverse perinatal outcomes in patients with gestational diabetes mellitus (GDM). **Methods**: Twenty-four (*n* = 24) patients with GDM and *n* = 29 age-matched pregnant women with normal glucose tolerance (NGT) were recruited at the third trimester of gestation. Clinical and biochemical parameters were collected. Serum levels of GDF-15, small dense low density lipoprotein cholesterol (sdLDL), interleukin 6 (IL-6), a Soluble Urokinase Plasminogen Activator Receptor (su-PAR) were measured by an enzyme-linked immunosorbent assay kit. Fetal ultrasound parameters, maternal, delivery, and perinatal outcomes, were assessed. **Results:** Serum GDF-15 did not differ between GDM and NGT (*p* = 0.286). However, in linear regression analysis, a significant negative association was observed between GDF-15 and fetal weight percentile at the third trimester, only in patients with GDM (*p* = 0.013), even after adjustment for age and pre-pregnancy BMI (*p* = 0.029). GDF-15 positively associated with IL-6, adjusting for pre-pregnancy BMI (*p* = 0.047). Pregnant women with adverse perinatal outcomes had higher levels of GDF-15 (*p* = 0.043). In the regression model, higher levels of GDF-15 were associated with an increased likelihood of adverse perinatal outcomes after adjustment for age and pre-pregnancy BMI (*p* = 0.044). **Conclusions**: Besides its action as regulator of inflammation, GDF-15 might have a possible protective role against hyperglycemia-related excessive fetal growth in GDM. GDF-15 circulating levels might also be related to adverse perinatal outcomes.

## 1. Introduction

Growth differentiation factor 15 (GDF-15), otherwise designated as macrophage inhibitory cytokine-1 (MIC-1), is a component of the transforming growth factor beta (TGF-β) superfamily [1]. This protein is highly expressed in response to cellular stress stimuli and demonstrated broad tissue expression patterns [2]. GDF-15 might also function as a crucial metabolic modulator in the pathophysiology of metabolic syndrome [3].

Gestational diabetes mellitus (GDM) refers to glucose intolerance that typically manifests during the second or third trimester of gestation [4]. GDM is associated with multiple adverse maternal and offspring outcomes, including but not limited to large for gestational age (LGA) fetuses, polyhydramnios, macrosomia, maternal hypertensive disorders, and various neonatal complications encompassing metabolic derangements (hypoglycemia, hyperbilirubinemia) [5,6]. Longitudinal studies have also demonstrated significant associations between GDM and subsequent maternal metabolic sequelae, including type 2 diabetes mellitus, metabolic syndrome, cardiovascular disease, and nephropathy [7,8,9].

The current literature examining the connection between GDF-15 and GDM remains limited in scope. An association between GDF-15 and glucose homeostasis parameters, including post-load glucose measurements and glycated hemoglobin, has been observed in women with GDM [10]. Circulating GDF-15 elevation was shown to predict microalbuminuria in subjects with GDM [11]. However, previous research that compared GDF-15 levels among women with GDM and normal glucose tolerance (NGT) led to conflicting results [12,13,14,15,16].

Despite the possible link between GDF-15 and the metabolic alterations in pregnancy, the association of GDF-15 with perinatal outcomes has been scarcely investigated so far. Specifically, one study failed to demonstrate any relationship between GDF-15 levels and perinatal outcomes in women with GDM and in control subjects [16].

The aim of this study was to explore the relationship between circulating levels of GDF-15 and metabolic/inflammatory parameters, as well as with adverse perinatal outcomes, in patients with GDM.

## 2. Patients and Methods

### 2.1. Study Subjects

Twenty-four (*n* = 24) patients with GDM and *n* = 29 age-matched pregnant women with NGT admitted to the outpatient clinics of Policlinico Umberto I, V Clinica Medica, “Sapienza” University Hospital of Rome were enrolled. The study was conducted in accordance with the Declaration of Helsinki, and the protocol was approved by the Hospital Ethics Committee of Policlinico Umberto I “Sapienza” University of Rome (project identification code 397-3435, date of approval 18 December 2014). Informed consent was obtained from all subjects involved in the study.

Current American Diabetes Association criteria for diagnosis of GDM were applied [4]. Women with GDM and control subjects were recruited in the third trimester of gestation. Only Caucasian subjects were included. Further exclusion criteria included multiple or induced pregnancies, the presence of inflammatory, infectious, or autoimmune diseases, polycystic ovarian syndrome, alcohol and drug abuse, and psychiatric diseases.

At recruitment, medical history was collected, including information about family history of T2D, parity, and GDM in a previous pregnancy. Physical and vital parameters, including pre-pregnancy weight and body mass index (BMI), third trimester weight, and systolic and diastolic blood pressure were obtained. The following laboratory parameters were measured: fasting plasma glucose (FPG), 1 h and 2 h PG after 75 g OGTT, third trimester FPG, glycated hemoglobin (HbA1c), total cholesterol [TC], triglycerides [TG], high density lipoprotein cholesterol [HDL-c], calculated low density lipoprotein cholesterol [LDL-c]), small dense LDL (sdLDL), 25-OH-vitamin D, GDF-15, interleukin 6 (IL-6), and the Soluble Urokinase Plasminogen Activator Receptor (suPAR).

Fetal ultrasound parameters, including abdominal circumference (AC), head circumference (HC), bi-parietal diameter (BPD), femur length (FL), humerus length (HL), estimated fetal weight (EFW), and fetal weight percentile at the third trimester were collected.

Maternal outcomes (insulin treatment for GDM, gestational hypertension/pre-eclampsia), neonatal sex and measures (birth weight, birth weight percentile, length, length percentile, head circumference, head circumference percentile), and delivery outcomes (gestational age at birth; preterm birth [<37 weeks of gestation], large for gestational age (LGA) [birth weight higher than the 90th percentile], macrosomia [birth weight, ≥4 kg], small for gestational age (SGA) [birth weight lower than the 10th percentile], fetal complications, Apgar score) were obtained. The variable adverse perinatal outcome was expressed as a composite of preterm delivery, LGA, SGA, a 1 min or 5 min Apgar score < 7, and fetal complications.

### 2.2. Laboratory Parameters

#### 2.2.1. Routine Parameters

Venous blood samples were collected after 8 h overnight fasting. Plasma glucose, HbA1c, TC, TG, HDL-c, and 25-OH-vitamin D were measured using commercially available kits.

#### 2.2.2. GDF-15

The GDF-15 assay was performed using a quantitative sandwich ELISA method (Quantikine QuickKit ELI-SA, R&D Systems, Minneapolis, MN, USA). A microplate is pre-coated with an anti-tag antibody. Standards and samples were added to the wells, followed by an antibody cocktail with an affinity-tagged monoclonal capture antibody and an enzyme-linked polyclonal detection antibody specific for human GDF-15. After removing unbound components, substrate solution was added, and color developed proportional to the amount of GDF-15 present. The reaction was then halted and the color intensity was measured.

A cut-off value of 556 pg/mL was determined, with a measurement range between 204 and 1146 pg/mL. The intra-assay and inter-assay coefficients of variation (CV) were below 3.0% and 10%, respectively.

#### 2.2.3. SdLDL

Serum levels of sdLDL were measured using a Sandwich ELISA kit (Novus, 12 Cambridge Science Park, Milton Road, Cambridge, CB4 0FQ, UK). The intra-assay and inter-assay coefficients of variation (CV) were <2.8% and <10%, respectively.

#### 2.2.4. IL-6

IL-6 levels were measured using the fully automated Elecsys system on a Cobas e801 platform (Roche Diagnostics, Basel, Switzerland). The Elecsys IL-6 immunoassay is standardized against first international standard 89/548 of the National Institute for Biological Standards and Control (NIBSC). The detection range of the assay was 1.5 to 5000 pg/mL, with a clinical cut-off value set at 7.0 pg/mL.

#### 2.2.5. SuPAR

SuPAR levels in serum samples were quantified using the suPARnostic ELISA kit (Biorbyt Ltd., 7 Signet Court, Swann’s Road, Cambridge, CB5 8LA, UK). The intra-assay and inter-assay CV were <2.7% and <10%, respectively.

### 2.3. Statistical Analysis

Mean values ± standard deviations were displayed for continuous variables. Categorical variables were expressed as frequencies. The normality of the variables was checked using the Kolmogorov–Smirnov test and visual methods. Differences between the groups were tested via an unpaired sample *t*-test when continuous variables had normal distribution. Means of skewed continuous variables were compared using the Mann–Whitney U test. Differences between the groups in categorical variables were evaluated via the Chi-Square test. Pearson’s correlation analysis was performed to examine the association between variables of interest. Univariable and multivariable linear regression analyses were performed to test the association of the variables of interest with GDF-15, adjusting for confounders. The association between circulating levels of GDF-15 and the variable adverse perinatal outcome (a composite of preterm delivery, LGA, SGA, a 1 min or 5 min Apgar score lower than 7, and fetal complications) was tested by a logistic model. A multivariable regression model was built to test for the association of GDF-15 with the variable adverse perinatal outcome, adjusting for the covariates’ age and pre-gestational BMI. A *p*-value < 0.05 was considered statistically significant. Statistical analysis was performed with IBM SPSS Statistics software version 23 (Chicago, IL, USA).

## 3. Results

Clinical parameters and laboratory measures of patients with GDM and the control group are displayed in Table 1. Table 2 and Table 3 show fetal ultrasound parameters and pregnancy outcomes, respectively.

Patients with GDM had significantly higher pre-pregnancy BMIs (GDM 26.7 ± 5.1 vs. NGT 23.0 ± 3.6 Kg/m^2^, *p* = 0.004), FPG at OGTT (GDM 87 ± 12.2 vs. NGT 78.3 ± 2.9 mg/dL, *p* = 0.009), 1 h PG (GDM 176.9 ± 31.3 vs. NGT 131.0 ± 21.4 mg/dL, *p* = 0.011), and 2 h PG (GDM 145.9 ± 29.6 vs. NGT 102.0 ± 20.2 mg/dL, *p* = 0.010) at OGTT, as well as third trimester FPG (GDM 82.6 ± 10.7 vs. NGT 71.7 ± 8.1 mg/dL, *p* < 0.001), than NGT subjects (Table 1). A first-degree family history of T2D was significantly more frequent among GDM patients than in the NGT group (GDM 45.8% vs. NGT 17.2%, *p* = 0.024), as well as a history of GDM in a previous pregnancy (GDM 22.7% vs. NGT 0%, *p* = 0.007). Weight increase was significantly lower in GDM than NGT (GDM 8.1 ± 6.0 kg vs. NGT 11.4 ± 4.4 kg, *p* = 0.037) (Table 1).

Circulating levels of GDF-15 were not different between GDM and NGT (GDM 1217.4 ± 155.8 pg/mL vs. NGT 1272.4 ± 193.4 pg/mL, *p* = 0.286). However, in linear regression analysis, a significant negative association was observed between GDF-15 and fetal weight percentile at the third trimester, only in patients with GDM (ß −0.09 [−0.15, −0.02] 95% CI, *p* = 0.013), not in NGT (ß 0.01 [−0.03, 0.06] 95% CI, *p* = 0.491). The inverse association between GDF-15 and fetal weight percentile in case subjects, although mildly attenuated, persisted significantly even after adjustment for age and pre-pregnancy BMI in the multivariable model (ß −0.09 [−0.16, −0.01] 95% CI, *p* = 0.029). Circulating levels of sdLDL, IL-6, and suPAR were not different between GDM and NGT (respectively, GDM 13.3 ± 2.2 vs. NGT 12.6 ± 2.6 mmol/L, *p* = 0.352; GDM 5.56 ± 2.04 vs. NGT 7.63 ± 9.88 pg/mL, *p* = 0.363; GDM 4.14 ± 1.80 vs. NGT 3.41 ± 1.93 ng/mL, *p* = 0.188). A significant positive correlation was observed between GDF-15 and IL-6 in the whole population (r = 0.296, *p* = 0.048). In the linear regression model, IL-6 significantly predicted GDF-15 levels (ß 7.06 [0.06, 14.06] 95% CI, *p* = 0.048), even after adjustment for pre-pregnancy BMI (ß 7.18 [0.11, 14.26] 95% CI, *p* = 0.047).

In the whole sample, the frequency of adverse perinatal outcomes was 24.4% without significant differences between GDM and NGT (GDM 17.6% vs. NGT 28.6%, *p* = 0.408). The group of pregnant women reporting adverse perinatal outcomes had significantly higher levels of GDF-15 (1352.4 ± 199.6 pg/mL vs. 1220.1 ± 167.1 pg/mL, *p* = 0.043). In the univariable regression model, higher levels of GDF-15 were associated with an increased likelihood of adverse perinatal outcomes (OR 1.004 [1.000–1.009] 95% CI, *p* = 0.044). In the multivariable model, GDF-15 values were independent predictors of adverse perinatal outcomes, after adjustment for age and pre-pregnancy BMI (OR 1.005 [1.000–1.009] 95% CI, *p* = 0.044) (Table 4).

## 4. Discussion

In this pilot cross-sectional study, circulating levels of GDF-15 did not significantly differ in patients with GDM and in control subjects at the third trimester of gestation. Previous studies comparing GDF-15 levels in GDM and NGT had yielded inconsistent results. Specifically, some authors showed greater GDF-15 levels in GDM patients than in control subjects in late pregnancy [12,14,15,16]. Conversely, in the third trimester, Jacobsen et al. observed significantly increased GDF-15 levels only in patients with pre-gestational type 1 diabetes, not in women with GDM, compared to the control group [13]. Battarbee et al. did not report any significant disparities in GDF-15 levels between subjects with previous GDM and controls 5–10 years after delivery [17]. Although a certain effect of low sample sizes cannot be excluded, the heterogeneity of the enrolled populations (i.e., different ethnic groups), together with the different diagnostic criteria adopted for GDM diagnosis or exclusion, might have contributed to the contrasting results between studies. In light of this, even though a possible link between GDF-15 and GDM has been postulated, whether GDF-15 might serve as a biomarker of disease is still a matter of debate and further studies with prospective design are necessary to confirm this association.

Although a clear link between circulating levels of GDF-15 and the presence of GDM did not emerge in this study, GDF-15 was significantly and inversely correlated to fetal growth only in patients with GDM. Of note, higher serum GDF-15 was significantly predictive of lower fetal weight percentile, independently of confounding factors, such as maternal age and pre-pregnancy BMI. Prior preclinical and clinical research has found a putative link between GDF-15 concentration and body weight [18,19,20]. Specifically, circulating levels of GDF-15 were found to be higher both in obese rats and in mice than in lean controls [18,20]. Preclinical findings were further confirmed by evidence from clinical studies reporting a positive correlation between GDF-15 and body weight, as well as with fat mass, in obese patients, independently of age and sex [18,19]. However, the underlying mechanisms that might explain this link are not well characterized and seem to be rather complex. Indeed, an inverse correlation between GDF-15 and BMI has also been described, suggesting that higher levels of this factor might associate with reduced BMI in non-obese patients [21]. In light of this, a valuable hypothesis beyond the controversial connection between GDF-15 and body weight is that the increase in GDF-15 is likely an adaptive mechanism against obesity, rather than a causative factor.

GDF-15 is expressed in gestational tissues, such as the placenta and the fetal membranes, and its release can be induced by high blood glucose, acting as a protective agent against glucose homeostasis imbalance [15,22]. Excessive fetal growth and macrosomia are well-recognized consequences of fetal exposure to high maternal glucose [23]. In animal studies, GDF-15 administration or overexpression led to food intake reduction, body weight decrease, and improvement in glucose tolerance, indicating a beneficial function of GDF-15 in glycolipid metabolism [24]. In this study, higher GDF-15 levels were related to lower fetal growth percentiles in the third trimester, suggesting a putative protective function of GDF-15 against hyperglycemia-induced excessive fetal growth in GDM. The fact that the inverse association of GDF-15 with fetal growth percentiles was not observed in the NGT group might be explained by the absence of the hyperglycemic state in physiological pregnancy. It might be hypothesized that in a normoglycemic context the possible protective role of GDF-15 against excessive fetal growth might not emerge. Interestingly, in line with these results, Diaz et al. reported an inverse association of circulating GDF-15 with ponderal index at birth in a cohort of appropriate and small for gestational age infants [25]. Although future studies should confirm these findings, high levels of GDF-15 might promote a negative energy balance in the fetus, thus associating with lower fetal and neonatal growth indices. Even though larger studies with longitudinal design are mandatory to shed more light on this question, considering the emerging evidence about a consistent relationship between circulating levels of GDF-15 and fetal growth indices, GDF-15 is a promising candidate biomarker for growth estimate in pregnancy complicated by GDM. Further confirmation of the potential role of GDF-15 as a predictor of fetal growth in GDM might have important clinical implications, given the current lack of reliable predictive biochemical markers for fetal growth and macrosomia in GDM pregnancy.

In this study, a significant positive association between serum GDF-15 and IL-6 was observed. Non-complicated pregnancy is typically hallmarked by an altered inflammatory profile compared to the non-pregnant state. The intricated immune regulation in pregnancy, which involves both innate and adaptive responses, results in the development of a systemic low-grade inflammatory state, ensuing from a fine balance between pro- and anti-inflammatory cytokines [26]. Chronic inflammation seems to significantly contribute to the development of insulin resistance, both in pregnancy and in non-pregnant condition. IL-6 is a pro-inflammatory cytokine released mainly by the immune cells, the adipocytes, and the endothelial cells, whose increased levels have been consistently linked to insulin-resistance and type 2 diabetes [27]. IL-6 is also secreted by the placenta, possibly contributing to the inflammatory state implicated in the onset of insulin-resistance during pregnancy [28]. Some studies have observed elevated levels of IL-6 in patients with GDM, whereas others have not confirmed this finding [29]. Of note, an increased gene expression of GDF-15 was shown to be positively correlated to high expression of genes contributing to inflammation in insulin target tissues [30]. Robust evidence has revealed that GDF-15 might exert anti-inflammatory actions, supporting the hypothesis that its elevation in inflammatory conditions is a compensatory response [1]). Of note, GDF-15 overexpression or administration significantly lowered circulating and tissue pro-inflammatory markers, including IL-6 [21]. To date, research exploring the correlation between GDF-15 concentration and pro-inflammatory cytokines levels in pregnancy is far limited. Interestingly, in line with these results, in a previous cross-sectional study involving patients with GDM and NGT, GDF-15 positively correlated with IL-6 [12]. It has been observed that GDF-15 may act as a regulator of macrophage activation. Specifically, GDF-15 release by macrophages is stimulated by pro-inflammatory cytokines, such as IL-6, secreted by activated macrophages, suggesting a role as a protective factor against the enhancement of the inflammatory response [31]. Considering that pro-inflammatory cytokines are also key contributors to the development of insulin resistance, the compensative increase in GDF-15 levels in response to IL-6 elevation might counteract this phenomenon, with a potential impact on clinical outcomes. In addition, inflammation is a well-established key contributor to the development of cardiovascular disease [32]. A growing body of evidence indicates that women with GDM and their offspring are exposed to lifelong increased cardiometabolic risk [8].

There is limited evidence about suPAR circulating levels in patients with GDM. Increased suPAR serum concentration has been described in T2D patients [33]. To the best of our knowledge, only one study evaluated circulating suPAR in patients with GDM, reporting a lower concentration in pregnant women who later developed GDM at 24–28 weeks [34]. In this study, suPAR levels, which were measured in late pregnancy, were comparable among GDM and NGT women. Future studies including larger populations are therefore needed to evaluate suPAR levels across different pregnancy stages and their possible link with GDM and pregnancy outcomes.

The association of GDF-15 levels with GDM-related worse perinatal outcomes has been poorly investigated so far. In this study, the group of pregnant women developing adverse perinatal outcomes had significantly higher levels of GDF-15. Of note, in the multivariate model, GDF-15 was a significant predictor of adverse perinatal outcomes, after adjustment for age and pre-pregnancy BMI. These findings suggest a possible link of this factor with pregnancy complications. Conversely, Yakut et al. previously had not observed any statistically significant relationship between GDF-15 and neonatal intensive care unit admissions in patients with GDM [16]. One reason for the discrepancy between studies may lie in the different pregnancy outcomes evaluated. Herein, adverse perinatal outcomes were analyzed as a composite of preterm delivery, LGA, SGA, pathological Apgar score, and fetal complications. Differently, Yakut et al. [16] evaluated birth weight, pathological Apgar score, primary cesarean section, tracing alterations, and neonatal intensive care unit admissions as single outcomes. In light of the conflicting results, future studies with longitudinal design and adequately powered to investigate the association of circulating GDF-15 with adverse perinatal outcomes in GDM are therefore required. Although the underlying mechanisms behind this possible association are yet to be determined, higher levels of GDF-15 in complicated pregnancy might be related to its compensative release in inflammatory and dysmetabolic states, which have been linked to the development of adverse perinatal outcomes [28]. Overall, these data suggest that measuring GDF-15 levels in complicated pregnancy may have relevant clinical implications, potentially helping identify subgroup of women with increased short- and long-term complications. However, whether GDF-15 may serve as a biomarker of adverse pregnancy outcomes remains to be determined in future studies.

## 5. Limitations and Future Research Directions

This study has several limitations. Firstly, this is a pilot study that enrolled a limited number of subjects. Although the exploratory nature of this study does not allow to draw firm conclusions, cross-sectional analysis might help identify associations between variables, which need further confirmation in adequately powered studies. GDF-15 was measured only at the third trimester of pregnancy, after the GDM screening test. Thus, changes in its concentration across different stages of gestation were not investigated. It is worth pointing out that GDF-15 levels may show different trends in various stages of pregnancy, and a single detection in the third trimester might not fully reveal how GDF-15 relates to the metabolic changes, as well as to fetal growth trajectory modifications throughout the entire gestation, both in physiology and in pathological conditions. Furthermore, in the multivariate linear regression model testing the association of GDF-15 with fetal growth and with IL-6, as well as in the regression model evaluating the relationship between GDF-15 and adverse perinatal outcomes, there was insufficient power to fully adjust for confounders other than age and pre-pregnancy BMI. Despite the acknowledged limitations, these results support the conclusion that GDF-15 might be connected to fetal growth in GDM pregnancy. Studies with a larger sample size are therefore needed to confirm this association and to clearly identify the underlying mechanisms. Specifically, whether GDF-15 is a direct contributor to fetal growth or just a biomarker should be evaluated in longitudinal investigations. Importantly, future research should reveal whether the correlation between GDF-15 and fetal growth is mediated by the action GDF-15 as a regulator of blood glucose levels.

To date, there is very limited evidence about GDF-15 levels in the context of pregnancy complications of GDM. Larger studies with fully adjusted models should explore whether short-term adverse outcomes of GDM could be effectively predicted by GDF-15 levels. Of note, given the impact of long-term complications of GDM, such as the development of type 2 diabetes and cardiovascular disease in the mothers and in the offspring, longer observations would allow for evaluating even longer-term outcomes of GDM pregnancy. In addition, whether the predictive role of GDF-15 on adverse events might be explained by its compensative increase in a pro-inflammatory context is an intriguing question for future research.

## 6. Conclusions

In conclusion, in this pilot study, an inverse association between circulating GDF-15 levels and fetal growth in late pregnancy was observed in patients with GDM, suggesting a possible protective effect of GDF-15 against hyperglycemia-related excessive fetal growth. In addition, GDF-15 levels directly correlated with IL-6 levels, indicating a putative action in contrasting inflammation. Finally, a link between GDF-15 and adverse perinatal outcomes emerged. Future studies with longitudinal design are needed to shed light on these associations.

## Figures and Tables

**Table 1:** Clinical and laboratory parameters of the study population.

Parameter	GDM (*n* = 24)	NGT (*n* = 29)	*p*-Value
Age (years)	35 ± 4.7	35.2 ± 5.5	0.908
Gestational age (weeks)	36.4 ± 1.2	37.0 ± 1.1	0.230
Pre-pregnancy BMI (Kg/m^2^)	26.7 ± 5.1	23.0 ± 3.6	0.004 *
Weight increase (kg)	8.1 ± 6.0	11.4 ± 4.4	0.037 *
T2D family history (%)	45.8	17.2	0.024 *
Previous GDM (%)	22.7	0	0.007 *
Multiparity n. (%)	87.5	75.9	0.281
Previous abortion n (%)	65.2	50.0	0.275
SBP (mmHg)	111.5 ± 11.9	111.0 ± 10.7	0.899
DBP (mmHg)	71.25 ± 9.7	68.7 ± 6.1	0.373
FPG at OGTT (mg/dL)	87 ± 12.2	78.3 ± 2.9	0.009 *
1 h PG at OGTT (mg/dL)	176.9 ± 31.3	131.0 ± 21.4	0.011 *
2 h PG at OGTT (mg/dL)	145.9 ± 29.6	102.0 ± 20.2	0.010 *
Third trimester FPG (mg/dL)	82.6 ± 10.7	71.7 ± 8.1	<0.001 *
HbA1c (%)	5.2 ± 0.5	5.1 ± 0.2	0.350
TSH (µUI/mL)	2.1 ± 1.6	2.6 ± 1.0	0.251
TC (mg/dL)	269.5 ± 60.9	288.4 ± 74.7	0.382
HDL-c (mg/dL)	70.4 ± 13.2	72.1 ± 13.7	0.683
LDL-c (mg/dL)	163.3 ± 52.0	168.6 ± 69.1	0.805
TG (mg/dL)	248.4 ± 113.5	246.9 ± 93.4	0.961
sdLDL (mmol/L)	13.3 ± 2.2	12.6 ± 2.6	0.352
25-OH vitamin D (ng/mL)	27.9 ± 11.4	33.4 ± 14.3	0.164
eGFR CKD-EPI (mL/min)	122.9 ± 8.3	121.4 ± 11.3	0.611
GDF-15 (pg/mL)	1217.4 ± 155.8	1272.4 ± 193.4	0.286
IL-6 (pg/mL)	5.56 ± 2.04	7.63 ± 9.88	0.363
SuPAR (ng/mL)	4.14 ± 1.80	3.41 ± 1.93	0.188

* *p*-Value < 0.05; BMI: body mass index; T2D: type 2 diabetes; GDM: gestational diabetes mellitus; SBP: systolic blood pressure; DBP: diastolic blood pressure; HbA1c: glycated hemoglobin; TSH: thyroid stimulating hormone; FPG: fasting plasma glucose; OGTT: oral glucose tolerance test; PG: plasma glucose; TC: total cholesterol; HDL-c: high density lipoprotein-cholesterol; LDL: low density lipoprotein-cholesterol; TG: triglycerides; sdLDL: small dense LDL; eGFR: estimated glomerular filtration rate; GDF-15: growth differentiation factor-15; IL-6: interleukin-6; SuPAR: Soluble Urokinase Plasminogen Activator Receptor.

**Table 2:** Fetal ultrasound parameters.

Parameter	GDM (*n* = 24)	NGT (*n* = 29)	*p*-Value
Gestational age (weeks)	33.7 ± 2.8	33.6 ± 2.3	0.927
AC (mm)	301.1 ± 32.8	295.9 ± 26.6	0.546
HC (mm)	299.2 ± 39.3	307.9 ± 22.2	0.403
BPD (mm)	86.0 ± 5.6	85 ± 5.9	0.529
FL (mm)	67.1 ± 4.9	64.8 ± 4.9	0.118
HL (mm)	60.2 ± 4.9	56.6 ± 6.6	0.074
EFW (g)	2407.9 ± 610.5	2301.3 ± 533.4	0.500
FW percentile	61.6 ± 25.4	51.9 ± 18.7	0.146

Abdominal circumference (AC), head circumference (HC), bi-parietal diameter (BPD), femur length (FL), humerus length (HL), estimated fetal weight (EFW), fetal weight (FW).

**Table 3:** Pregnancy outcomes.

Parameter	GDM (*n* = 24)	NGT (*n* = 29)	*p*-Value
MATERNAL OUTCOMES			
Insulin treatment (%)	37.5	-	-
Gestational hypertension (%)	17.4	3.4	0.090
NEONATAL PARAMETERS			
Sex (M,%)	50.0	64.0	0.375
Weight (g)	3227 ± 407	3295 ± 366	0.526
Weight percentile	43.9 ± 27.8	45.8 ± 29.4	0.812
Length (mm)	48.9 ± 1.6	48.8 ± 1.8	0.897
Length percentile	53.9 ± 15.0	48.9 ± 12.6	0.264
HC (cm)	34.6 ± 1.4	34.6 ± 1.5	0.936
HC percentile	25.4 ± 10.8	23 ± 9.47	0.469
DELIVERY OUTCOMES			
Gestational age at birth (weeks)	38.4 ± 0.9	38.8 ± 0.8	0.093
Preterm birth (%)	4.3	0	0.257
Macrosomia (%)	0	3.4	0.369
LGA (%)	4.3	6.9	0.695
SGA (%)	4.3	16.8	0.251
Apgar 1 min	8.7 ± 0.5	8.4 ± 0.8	0.088
Apgar 5 min	9.7 ± 0.5	9.5 ± 0.6	0.180
Apgar 1 min or 5 min < 7 (%)	3.4	0	0.369
Fetal complications (%)	0	4.8	0.261
Adverse perinatal outcome (%)	17.6	28.6	0.408

HC: head circumference. Adverse perinatal outcome was defined as a composite of preterm delivery, LGA, SGA, a 1 min or 5 min Apgar score < 7, and fetal complications.

**Table 4:** Logistic regression model for adverse perinatal outcomes.

	Multivariable Analysis		
	OR	95% CI	*p*-Value
GDF-15	1.005	(1.000–1.009)	0.044 *
Age	0.974	(0.840–1.130)	0.729
Pre-pregnancy BMI	1.039	(0.879–1.229)	0.652

* *p* < 0.05; GDF-15: growth differentiation factor-15; BMI: body mass index.

## Data Availability

The data presented in this study are available on request from the corresponding author (the data are not publicly available due to ethical restrictions).
